# Comparing the Clinical and Radiological Outcome of Femoral Neck System Versus Dynamic Hip Screw in Femoral Neck Fractures

**DOI:** 10.7759/cureus.96595

**Published:** 2025-11-11

**Authors:** Abhijeet Kunwar, Birju Manjhi, Prince Maheshwari, Utkarsh Kumar, Satyam Kumar, Rajeev Kumar Ranjan

**Affiliations:** 1 Trauma Orthopaedics, Trauma Centre, Super Speciality Hospital, Varanasi, IND; 2 Orthopaedics, Institute of Medical Sciences, Banaras Hindu University, Varanasi, IND; 3 Orthopaedics and Traumatology, Institute of Medical Sciences, Banaras Hindu University, Varanasi, IND

**Keywords:** dynamic hip screw, femoral neck system, fracture neck of femur, minimally invasive procedure, trauma

## Abstract

Purpose

Since its introduction in 1964, the dynamic hip screw (DHS) has remained a mainstay in treatment, offering reliable fixation, high union rates, and early weight-bearing. However, its invasive nature poses a risk of compromising the femoral head's blood supply. On the other hand, multiple cancellous cannulated screw (CCS) allow for a minimally invasive approach but often limit early mobilization. In 2018, DePuy Synthes, Switzerland, introduced the femoral neck system (FNS), promising stability of the fixation equivalent to DHS while maintaining the minimally invasive nature of the procedure. This study aims to compare the clinical and radiological outcomes of DHS and FNS.

Methodology

A prospective, comparative study was conducted among 120 patients with femoral neck fractures, divided randomly into two groups to remove confounding: Group A (n = 60) - treated with FNS; Group B (n = 60) - treated with DHS. Intraoperative parameters assessed included duration of surgery, blood loss, number of C-arm shoots, and length of incision. Postoperative outcomes were evaluated using the Harris Hip Score, radiological union, and complications such as AVN, varus malunion, femoral neck shortening, and screw back-out were noted.

Results

The study revealed statistically significant advantages of FNS over DHS in intraoperative parameters: mean duration of surgery: FNS - 28.9 min vs. DHS - 47.86 min (p < 0.05), mean blood loss: FNS - 12.53 mL vs. DHS - 38.91 mL (p < 0.05), mean C-arm exposures: FNS - 17.68 vs. DHS - 23.55 (p < 0.05), mean incision length: FNS - 4.76 cm vs. DHS - 11.53 cm (p < 0.05). Postoperative outcomes showed a comparable union rate (16.13 FNS vs. 16.03 DHS), but the Harris Hip Score was higher with FNS 63.76, 76.55, and 88.88 vs. DHS 60.96,76.21, and 88.01 at 4, 12, and 24 weeks, respectively. Early results and final outcome are in favor of FNS (p < 0.05). A total of 32 complications were observed: 11 in FNS vs. 21 in DHS.

Conclusion

FNS significantly outperformed DHS in terms of operative efficiency, reduced blood loss, smaller surgical exposure, and fewer complications. These findings suggest that the FNS is a superior and more suitable alternative for the treatment of femoral neck fractures, especially in younger patients.

## Introduction

Fracture of the femoral neck is a long-standing challenge for orthopedic surgeons due to high rates of associated complications. Because of these complications, back in 1934, Speed [1] labelled femoral neck fracture (FNF) as the “Unsolved Fracture.” These complications are due to the precarious blood supply of the femoral head, fracture geometry and unstable fracture patterns, intra-capsular location of fracture, absent cambium layer, poor surgical technique, posteromedial comminution, and difficulty in reduction. Previously, the majority of patients, i.e., around 70% patients with FNF, belonged to the age of more than 65 years [2], who have sustained injury due to low-energy trauma like a trivial fall, and hemiarthroplasty/total arthroplasty has been an established solution for these patients. In recent decades, there has been an increase in the trend of FNF among the young population due to high-velocity trauma, imposing a greater challenge to surgeons as this trauma produces displaced and unstable fracture patterns [3]. Achieving union with minimal complications and associated comorbidity is of prime importance due to the high physical demand of these patients during their remaining life span; hence, young adults require reliable fixation and union. Over the past century, several methods and devices were invented for the fixation of “Unsolved Fracture” like multiple pinning, wires, cancellous cannulated screw (CCS), lag screw, flagged nails, angled blade plate, proximal femur locking plates [4], but none of them were universally accepted as union in FNF is difficult to achieve. In 1964, Clawson [5] introduced the dynamic hip screw (DHS), which offers reliable stabilization and fixation and has a good outcome. Several advancements were made in DHS, such as the use of an angled plate, insertion of a helical blade, use of an anti-rotation screw, etc. Thus, for the past 50 years, DHS has remained the cornerstone in the treatment of the fractured neck of the femur [6]. Despite all the advantages, DHS is also associated with some difficulties due to the invasive nature of the surgery that poses the risk of compromising the blood supply of the head, leading to a higher degree of avascular necrosis (AVN) [7]. Use of multiple CCS in different patterns is another popular method for the fixation of FNF, which is minimally invasive, yet their role is limited as the procedure is technically more demanding, requires a high degree of radiation exposure, and the patient needs a long period of immobilization following surgery [8].

In 2018, DePuy Synthes, Switzerland [9], introduced the femoral neck system (FNS) that comprises a locking plate, a neck bolt, and an anti-rotation screw. This device is claimed to provide angular, rotational, and torsional stability and can be fixed with a minimally invasive method [10]. Comprehensive bio-mechanical studies conducted on human cadavers show good efficacy of FNS as an effective alternative to DHS [10]. Following the introduction, an increase in the trend of FNS fixation and its popularity among the upcoming orthopedic surgeons led us to conduct this study, where we have compared the outcome of these two traditional and novel devices to find which one is more suitable for fixing a fractured femoral neck.

## Materials and methods

For the abovementioned comparison, we have conducted a prospective, randomized study in the Department of Orthopedics, Institute of Medical Sciences (IMS), Banaras Hindu University (BHU) from March 2022 to March 2025 among 120 patients. All patients reported to our center with FNF were informed about the study, and patients with positive consent to participate in the study aged between 18 and 65 years with isolated FNF were included in the study. Patients with pathological fractures, fractures older than one week, patients with pre-existing arthritic hip conditions, poly-trauma patients, patients with open fractures, and patients with on-going local or systemic infection were excluded from the study. Selected patients were divided into two groups: Group A (n = 60) patients treated with FNS and Group B (n = 60) patients treated with DHS (as shown in the figures in the Results section). Randomization was done by the odd and even method to remove confounding from each group. Age, gender, laterality, mode of injury, date of injury, history of any drug allergies, and fractures classification by Pauwels’ [11] and Garden’s [12] method of classification were recorded preoperatively. All patients were operated under similar conditions, i.e., under spinal anesthesia on a fracture table under fluoroscopic guidance. Procedures were performed by experienced orthopedic surgeons, and standard surgical steps were followed in both groups [13,14]. Reduction and implant position were continuously monitored under the C-arm during surgery. Intraoperatively, the duration of surgery (in minutes), the length of incision (in centimeters), the blood loss (gauze visual analogue method) [15], and C-arm exposure (number of C-arm shoots) were noted. One antibiotic shot was given 30 minutes before surgery, and postoperatively, two shots of IV antibiotics were given, and patients were discharged on oral antibiotics on postoperative day 2 after one dressing of the incision site. Patients were taught isometric quadriceps and ankle mobility exercises. Hip and knee bending started on the next day of the surgery. Patients were allowed partial weight bearing from three weeks after the surgery, and load on the operated limb was allowed as per the pain tolerance of the patient. Patients were called for follow-up at two weeks for suture removal, four weeks for a check X-ray, and monthly after that for a minimum duration of 12 months. X-rays were done at an interval of every four weeks till the union is achieved on X-ray, i.e., three united cortices out of four cortices in two orthogonal views. Full weight bearing is allowed once union is documented on X-ray. Harris Hip Score (HHS) [16] was calculated at an interval of four weeks, 12 weeks, and 24 weeks and was graded: 90-100 as excellent, 80-89 as good, 70-79 as moderate, and less than 70 as poor. During the follow-up, complications like AVN of the femoral head, non-union, varus malunion, femoral neck shortening, screw back-out, surgical site infection, etc., were recorded.

Statistical analysis

Data were recorded, and continuous variables were expressed as mean and standard deviation and compared using Student’s t-test [17], and categorical data were expressed in percentage and range and compared using the chi-square test. Results were considered significant with a p-value < 0.05.

## Results

The study was conducted among 120 patients, 60 patients in each group, with a mean age of 43 years (FNS group = 43.5 ± 12.36 years and DHS group = 42.58 ± 13.21 years). Patients were followed for a mean duration of 15.2 months (FNS = 15.2 months (12-24 months) and DHS = 15.23 months (12-24 months)). Among these patients, 75 (62.5%) were male, and 45 (38.5%) were female (FNS = 37 (61.66%) male and 23 (38.33%) female, DHS = 38 (63.33%) male and 22 (36.66%) female). A total of 67 (55.8%) patients had right-sided fractures, and 53 (44.2%) patients had left-sided fractures (FNS = 34 (56.6%) right and 26 (43.3%) left, and DHS = 33 (55%) right and 27 (45%) left). The majority of the patients, 86 (71.66%), have sustained trauma due to a road traffic accident, followed by 34 (28.33%) patients who have sustained trauma due to a fall. Demographic distribution of the patient in each group is as follows (Table 1).

**Table 1:** Demographic distribution and preoperative detailing of patients in both groups DHS, dynamic hip screw; FNS, femoral neck system; RTA, road traffic accident

	FNS		DHS		Total	
Mean age	43.5 ± 12.36 years		42.58 ± 13.21 years		43 years	
Gender	Male - 37 (61.66%)	Female - 23 (38.33%)	Male - 38 (63.33%)	Female - 22 (36.66%)	Male-75 (62.5%)	Female - 45 (38.5%)
Laterality	Right - 34 (56.6%)	Left - 26 (43.3%)	Right - 33 (55%)	Left - 27 (45%)	Right - 67 (55.8%)	Left - 53 (44.2%)
Mode of injury	RTA - 44 (73.33%)	Fall - 16 (26.66%)	RTA- 42 (70%)	Fall - 18 (30%)	RTA - 86 (71.66%)	Fall - 34 (28.33%)

A total of 67 (55.83%) patients belong to Pauwels’ [11] class II, followed by 43 (35.83%) in Pauwels’ class III, and 10 (8.33%) patients in class I (Table 2).

**Table 2:** Pauwels’ classification of the study subjects in the FNS and the DHS group DHS, dynamic hip screw; FNS, femoral neck system

Pauwels’ classification	FNS	DHS
Pauwels I	6 (10%)	4 (8.33%)
Pauwels II	33 (55%)	34 (56.66%)
Pauwels III	21 (35%)	22 (38.33%)
Total	60	60

According to Graden’s [12] classification (Table 3), the majority of patients belong to Garden’s index III, with 61 (50.83%) followed by Garden’s index II, with 34 (28.33%), then Garden’s index IV, with 16 (13.33%), and finally Garden’s index I, with nine (7.5%).

**Table 3:** Garden's classification of the study subjects in the FNS and the DHS groupDHS, dynamic hip screw; FNS, femoral neck system

Garden’s Index	FNS	DHS
Garden’s index I	5 (8.33%)	4 (6.66%)
Garden’s index II	18 (30%)	16 (26.6%)
Garden’s index III	29 (48.33%)	32 (53.3%)
Garden’s index IV	8 (13.33%)	8 (13.33%)
Total	60	60

Most of the patients, i.e., 63 (52.5%), were operated on three to four days after trauma, 38 (31.66%) patients were operated from zero to two days after trauma, and 19 (14.61%) patients were operated from five to seven days following trauma (Table 4).

**Table 4:** Injury to surgery interval of the patients in the FNS and the DHS group DHS, dynamic hip screw; FNS, femoral neck system

Injury to surgery interval	FNS	DHS
Zero to two days	19 (31.66%)	19 (31.66%)
Three to four days	32 (53.33%)	31 (51.66%)
Five to seven days	9 (15%)	10 (16.66%)
Total	60	60

Considering the intraoperative parameters, the mean duration of surgery was 28.9 ± 6.91 minutes for the FNS group, while it was 47.86 ± 8.73 minutes for the DHS group. Mean length of incision was 4.76 ± 0.98 cm and 11.35 ± 1.58 cm for FNS and DHS groups, respectively. Blood loss was measured by the gauze visual analogue method [15], and 12.53 ± 3.16 mL and 38.91 ± 9.91 mL were the mean recorded blood loss in the FNS and DHS groups, respectively. In the FNS group, the mean C-arm shoots were 17.68 ± 3.89 shoots, and in the DHS group, the mean C-arm shoots were 23.55 ± 3.97. For all four intraoperative parameters evaluated during studies, a significant difference is recorded in the mean value of data (Table 5).

**Table 5:** Intraoperative parameters and comparison of both study groups DHS, dynamic hip screw; FNS, femoral neck system

	FNS			DHS			p-value
	Mean	Range	SD	Mean	Range	SD	
Duration of surgery (min)	28.9	20-45	6.91	47.86	30-66	8.73	<0.05
Length of incision (cm)	4.76	4-8	0.98	11.35	10-17	1.58	<0.05
Blood loss (mL)	12.53	10-20	3.16	38.91	25-60	9.91	<0.05
C-arm shoot (number of shoots)	17.68	10-33	3.89	23.55	16-38	3.97	<0.05

**Figure 1:**
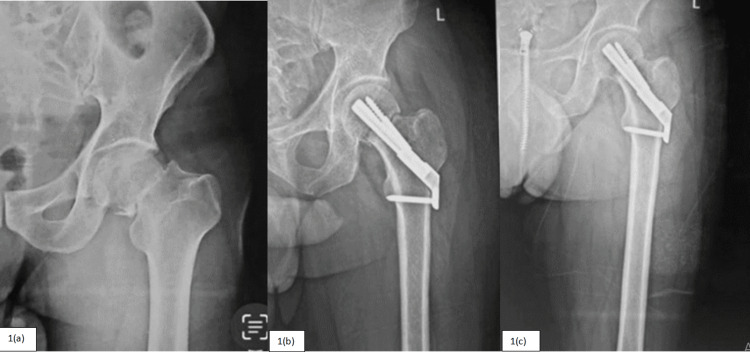
Study subject of group A patient treated with femoral neck system (a) - Preoperative. 1(b) - Immediate postoperative. 1(c) - Union at follow-up.

**Figure 2:**
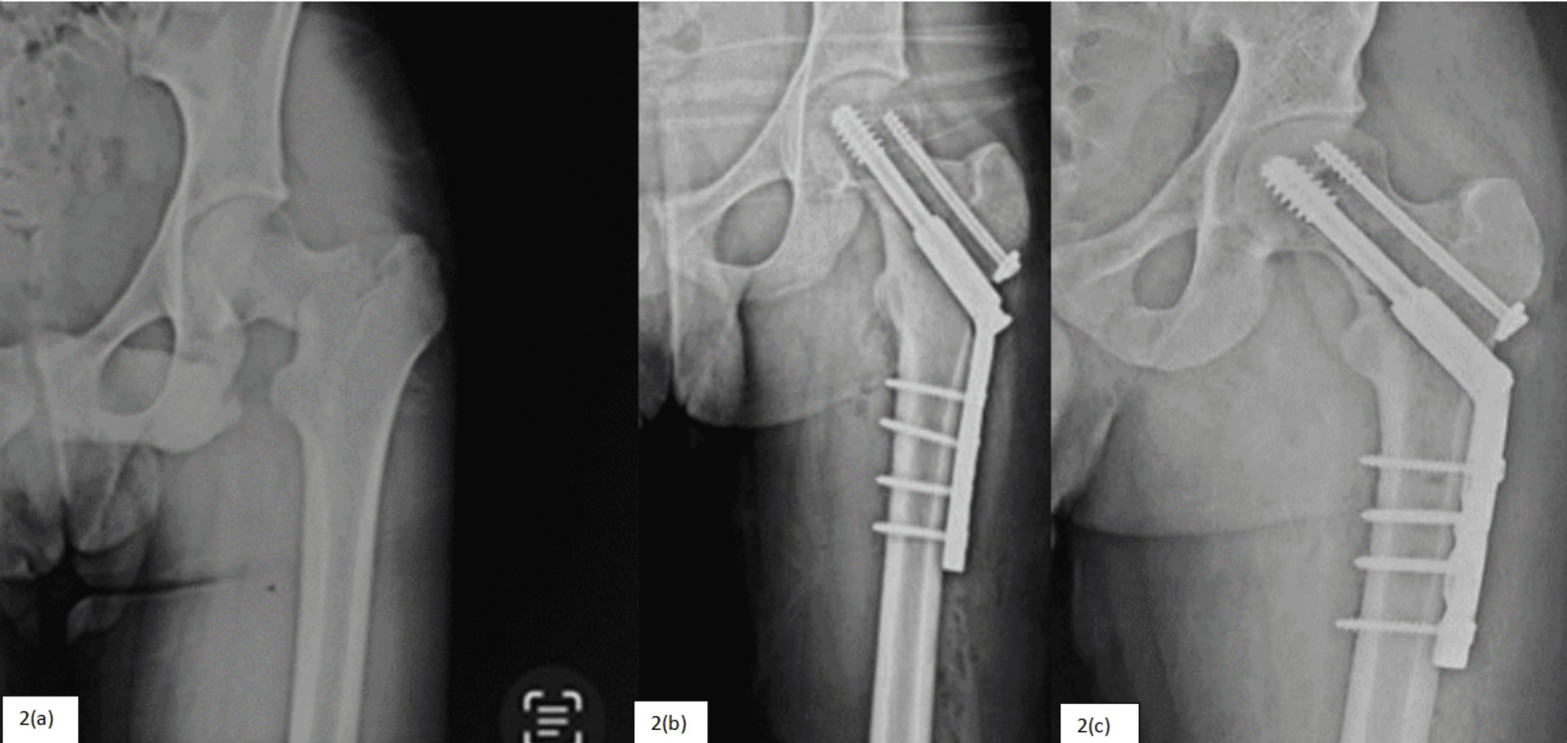
Study subject of group B patient treated with dynamic hip screw 2(a) - Preoperative. 2(b) - Immediate postoperative. 2(c) - Union at follow-up.

Patients were followed for a mean duration of 15.2 months (FNS = 15.18 months and DHS = 15.23 months). For the clinical assessment, HHS was calculated at 4, 12, and 24 weeks during the follow-up. For the FNS group, HHS found to be 63.76 ± 4.95, 76.55 ± 4.42, and 88.88 ± 2.73, and for the DHS group, it was 60.96 ± 5.59, 76.21 ± 4.54, and 88.01 ± 3.11, respectively, during each follow-up. Significant difference in the functional outcome was recorded at four and 24 weeks in favor of the FNS group. Mean union time on radiograph (three united cortices out of four in two orthogonal views) for the FNS group was 16.13 weeks (Figure 1) and for the DHS group was 16.03 weeks (Figure 2), and results were comparable in both groups. Follow-up results of the study are summarized below (Table 6).

**Table 6:** Follow-up details and comparison of both study groups DHS, dynamic hip screw; FNS, femoral neck system

	FNS			DHS			p-value
	Mean	Range	SD	Mean	Range	SD	
HHS at four weeks	63.76	50-70	4.95	60.96	50-70	5.59	<0.05
HHS at 12 weeks	76.55	65-85	4.42	76.21	65-85	4.54	0.09
HHS at 24 weeks	88.88	80-94	2.73	88.01	82-94	3.11	0.015 (<0.05)
Union time	16.13	12-24	1.83	16.03	12-24	1.78	0.37

Complications

**Figure 3:**
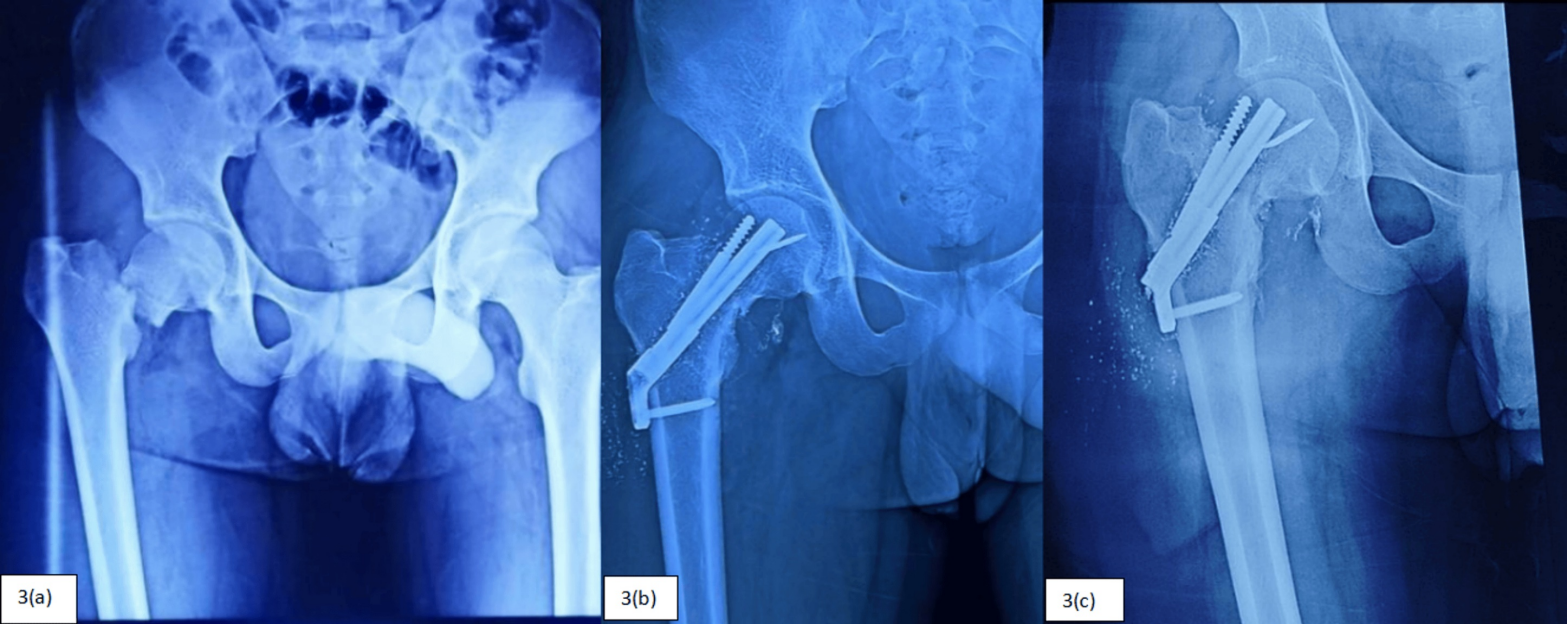
Patient treated with FNS showing intraoperative guide wire breakage and varus collapse in follow-up 3(a) - Preoperative. 3(b) - Immediate postoperative. 3(c) - Varus collapse in follow-up.

**Figure 4:**
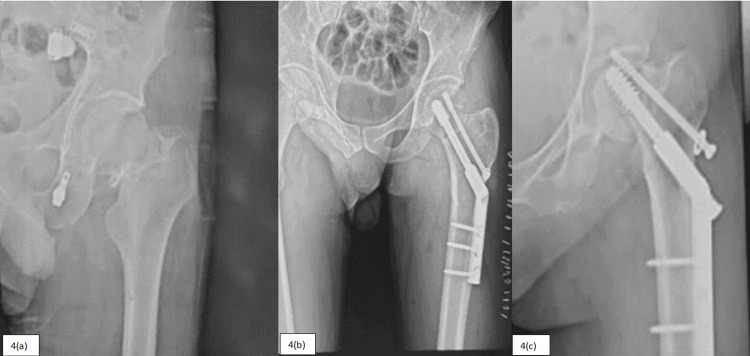
Patient treated with dynamic hip screw showing varus collapse and screw back out in follow-up 4(a) - Preoperative. 4(b) - Immediate postoperative. 4(c) - Varus collapse and screw backout in follow-up.

During the study, complications like AVN of the femoral head, neck shortening > 5mm, superficial surgical site infection, screw back-out, and varus collapse (Figures 3-4) were recorded, and the frequency of the complications is as follows (Table 7).

**Table 7:** Complications recorded during follow-up in each group AVS, avascular necrosis; DHS, dynamic hip screw; FNS, femoral neck system

Complication	FNS	DHS
Total	11	21
Superficial surgical site infection	1	4
Deep infection	0	0
AVN	1	3
Screw backout	1	6
Femoral neck shortening >5mm	2	3
Varus collapse	3	2
Non-union	2	3
Guide wire break	1	0

## Discussion

In 2019, Shehata et al. [18] concluded that for stable fracture patterns, both multiple CCS and sliding hip screw led to good outcomes with less intraoperative blood loss in the multiple CCS group, while in 2020, Şahin et al. [19] in their retrospective study comparing DHS with anti-rotation screw and conventional cannulated screw for FNFs concluded that DHS + anti-rotation screw shows superior outcome and less fluoroscopic exposure, hence a preferred treatment method.

Zhou et al. (2021) [20], in their study to compare FNS and multiple CCS, found that patients treated with FNS had lower postoperative pain, early mobilization without crutches, better HHS, and fewer complications, while He et al. [21] concluded that in young adults, similar fracture healing occurred in both the CCS and FNS group, with less fluoroscopic exposure and fewer complications in the FNS group.

In our study, while comparing the two groups, FNS and DHS, we have observed that the mean age of the study population was 43 years (FNS group = 43.5 ± 12.36 years and DHS group = 42.58 ± 13.21 years), which is somewhat consistent with the study conducted by Ge et al. [22], i.e., 50.4 ± 7.4 years for the FNS group and 48.2 ± 8.0 years for the DHS group, although the mean age of the study population in the study conducted by Schuetze et al. [23] was 69 ± 14 years. In our study, we have excluded the population older than 65 years, as hemiarthroplasty/total arthroplasty is a more suitable option for elderly patients than osteosynthetic devices. In our study 62.5%, i.e., 75 (62.5%) patients out of 120 study participants were male, which is similar to the study conducted by Ge et al. [22], in their observation 70.5% study participants were male in contrast with the study of Schuetze et al. [23], where in DHS group had only 50.9% and in FNS group only 47.8% patients were male, as in relatively younger population high energy trauma (road traffic accidents) is more common mechanism of injury [24] that primarily affect male population while in elderly age group trauma is caused due to trivial fall that predominantly affects female population, as osteoporosis is more common in female lead to poor bone quality. In our study, 86 patients, i.e., 71.66% have sustained trauma due to high velocity injury (road traffic accident). As per the fracture classification majority of the patients belongs to Pauwels’ II and III - 110 (91.66%) patients and Garden’s III and IV - 77 (64.16%) similar to the patients in the study of Ge et al. [22], where 86.3% patients belongs to Pauwels’ grades II and III and 87.3% patients belongs to Garden’s III and IV while in the study of Konrad et al. [23] where majority of patients were Pauwels’ II, i.e., 57.01% and Garden’s II 47.5% which represents that in older patients trivial trauma produce stable fracture patterns where in younger population high energy trauma produce unstable fracture patterns [25].

Duration of surgery is significantly less in the FNS group compared to the DHS group; operating time for the FNS group was 28.9 ± 6.91 min, and for the DHS group was 47.86 ± 8.73 min; these results were comparable to both Ge et al. [22] (FNS = 47.09 ± 9.19 min vs. DHS =52.90 ± 9.64 min) and Schuetze et al. [23] (FNS = 36.3±11.6 vs. DHS = 54.7±17.4 min). The scar length of incision for the FNS group was 4.76 ± 0.98 cm, and for the DHS group was 11.35 ± 1.58 cm. These results were consistent with the study of Niemann et al. [26], where the median scar length for FNS was 4.5 cm and for DHS was 9.5 cm. Similarly, intraoperative blood loss was significantly lower in the FNS group, i.e., 12.53 ± 3.16 mL and in the DHS group is 38.91 ± 9.91 mL, although comparable blood loss in the study of Ge et al. [22], is significantly less is the FNS group, i.e., 48.53 ± 10.69 mL (FNS) vs. 65.31 ± 17.91 mL (DHS), yet absolute higher value in the blood loss is attributed to use of a two-hole plate in their study compare to the use single whole plate in our study. Intraoperative radiation exposure is also significantly less in the FNS fixation compared to the DHS fixation (17.68 ± 3.89 shoots vs. 23.55 ± 3.97 shoots). Similar results were attributed in favor of FNS when compared to multiple CCS [19].

Patients were followed for a mean duration of 15.2 months, and during the postoperative follow-up time, the mean union time was comparable in both groups (FNS 16.13 ± 1.83 months vs. DHS 16.03 ± 1.78 months). The absolute value of the mean union time was higher when compared with the previously recorded mean union time. For the FNS, it was 12.43 ± 3.34 weeks in the study of Kenmegne et al. [27], and for the DHS, it was 12.28 ± 3.71 weeks in the study of Kulambi et al. [28]. Longer union time is attributed to the delay in weight bearing in the immediate postoperative period. Clinical outcome of the study represents early improvement in the patients operated with the FNS compared to the DHS (HHS at four weeks 63.76 ± 4.95 vs. 60.97 ± 5.59). Early recovery of the patients in the FNS group is due to the intraoperative advantages of FNS over DHS. A less invasive procedure can be done using a skin incision less than 5 cm if planned properly, significantly lesser surgical duration and less implant foot print on the lateral cortex lead to early recovery and lesser post op pain although the results become comparable at long-term follow up (HHS at 12 weeks FNS 76.55 ± 4.42 vs. DHS 76.21 ± 4.54) as the union rate is comparable in both the groups. At the follow-up period of 24 weeks, results were slightly in favor of the FNS group (HHS at 24 weeks 88.88 ± 2.73 vs 88.01 ± 3.11) (p-value = 0.015). These results were again consistent with the study of Ge et al. [22], where the clinical outcome of FNS was superior to DHS (92.3 ± 4.5 vs. 89.9 ± 4.9). In long-term follow-up, better results in the FNS group are due to the fewer long-term complications in the FNS group.

There were 11 (18.33%) complications recorded in the FNS group and 21 (35%) in the DHS group - five cases of superficial surgical site infection, one in the FNS and four in the DHS, all cases were treated with regular dressing and IV antibiotics, and the infection was resolved without the requirement of any extensive debridement or implant removal. More cases of SSI were seen in the DHS group due to the longer incision and more surgical duration [29]. Four cases of AVN of the femoral head were reported, one in the FNS group and three in the DHS group. One case in each group was treated with implant removal, core decompression, and bone grafting. The remaining two cases in the DHS group were treated with total hip arthroplasty (THA). Lower incidence of the AVN is due to the less invasive nature, preserved bone stock, and vascularity in the FNS group. Seven cases of screw backout were seen, one in the FNS group and 6 in the DHS group; a higher incidence in the DHS group is attributed to the cancellous collapse phenomenon that occurs in DHS. One case in the FNS group and two cases in the DHS group were treated with implant removal, two cases in the DHS group were managed conservatively as patients only had mild abductor lurch, and the remaining case in the DHS group was treated with THA. Five cases of femoral neck shortening were reported, and none of the patients had significant complaints in activity; hence, they were only managed conservatively. Five cases of non-union were reported, two in the FNS group and three in the DHS group, with two cases in each group. They were treated with revision surgery and bone grafting, while one patient in the DHS group underwent hemiarthroplasty. A total of five cases of varus collapse were reported: three in the FNS group and two in the DHS group. One patient in the FNS group had intraoperative guide wire breakage in the femoral head and also had varus collapse in the follow-up. This patient was treated with THA. One patient in the DHS group was treated with modular bipolar; the remaining patients were treated with valgus osteotomy and fixed with double-angle DHS.

## Conclusions

For more than half a century, DHS has remained the cornerstone for the management of FNFs, but in the past decade, the emergence of FNS has provided a more convenient yet reliable method of fixation for the FNFs, which is easy to use and produces more consistent results, promotes early mobilization and quicker recovery, and is associated with a lower rate of complications.
